# Biomarker treatment effects in two phase 3 trials of gantenerumab

**DOI:** 10.1002/alz.14414

**Published:** 2025-01-30

**Authors:** Tobias Bittner, Matteo Tonietto, Gregory Klein, Anton Belusov, Vittorio Illiano, Nicola Voyle, Paul Delmar, Marzia A. Scelsi, Susanna Gobbi, Erica Silvestri, Muhamed Barakovic, Antonio Napolitano, Christopher Galli, Maryam Abaei, Kaj Blennow, Frederik Barkhof

**Affiliations:** ^1^ Genentech, Inc. South San Francisco California USA; ^2^ F. Hoffmann‐La Roche Ltd Basel Switzerland; ^3^ Roche Products Limited Welwyn Garden City UK; ^4^ A4P Consulting Ltd. Sandwich UK; ^5^ Department of Psychiatry and Neurochemistry Institute of Neuroscience and Physiology the Sahlgrenska Academy at the University of Gothenburg Mölndal Sweden; ^6^ Clinical Neurochemistry Laboratory Sahlgrenska University Hospital Mölndal Sweden; ^7^ Paris Brain Institute ICM Pitié‐Salpêtrière Hospital Sorbonne University Paris France; ^8^ Department of Radiology & Nuclear Medicine Amsterdam UMC Vrije Universiteit Amsterdam the Netherlands; ^9^ UCL Queen Square Institute of Neurology and Centre for Medical Image Computing, Queen Square London UK

**Keywords:** Alzheimer's disease, amyloid beta, anti‐amyloid, biomarkers, blood‐based biomarkers, cerebrospinal fluid, gantenerumab, neurofilament light, phase 3, phosphorylated tau181, phosphorylated tau 217, positron emission tomography, tau, volumetric magnetic resonance imaging

## Abstract

**INTRODUCTION:**

We report biomarker treatment effects in the GRADUATE I and II phase 3 studies of gantenerumab in early Alzheimer's disease (AD).

**METHODS:**

Amyloid and tau positron emission tomography (PET), volumetric magnetic resonance imaging (vMRI), cerebrospinal fluid (CSF), and plasma biomarkers used to assess gantenerumab treatment related changes on neuropathology, neurodegeneration, and neuroinflammation over 116 weeks.

**RESULTS:**

Gantenerumab reduced amyloid PET load, CSF biomarkers of amyloid beta (Aβ)40, total tau (t‐tau), phosphorylated tau 181 (p‐tau181), neurogranin, S100 calcium‐binding protein B (S100B), neurofilament light (NfL), alpha‐synuclein (α‐syn), neuronal pentraxin‐2 (NPTX2), and plasma biomarkers of t‐tau, p‐tau181, p‐tau217, and glial fibrillary acidic protein (GFAP) while increasing plasma Aβ40, Aβ42. vMRI showed increased reduction in whole brain volume and increased ventricular expansion, while hippocampal volume was unaffected. Tau PET showed no treatment effect.

**DISCUSSION:**

Robust treatment effects were observed for multiple biomarkers in GRADUATE I and II. Comparison across anti‐amyloid antibodies indicates utility of p‐tau and GFAP as biomarkers of amyloid plaque removal while NfL and tau PET seem unsuitable as consistent indicators of clinical efficacy. vMRI might be confounded by non‐neurodegenerative brain volume changes.

**TRIAL REGISTRATION NUMBER (CLINICALTRIALS.GOV IDENTIFIER)**:

NCT03444870 and NCT03443973.

**Highlights:**

- Gantenerumab significantly reduced brain amyloid load.
- Tau positron emission tomography showed no treatment effect in a small subset of participants.
- Volumetric magnetic resonance imaging showed increased whole brain volume reduction under treatment while hippocampal volume was unaffected.
- Robust treatment effects on cerebrospinal fluid and plasma biomarkers were found, despite lack of clinical efficacy.

## 1 BACKGROUND

Alzheimer's disease (AD) is the most common cause of age‐related cognitive and functional impairment and dementia. Monoclonal antibodies targeting amyloid beta (Aβ) proteins of different species have been developed to treat people with AD and have shown mixed results in phase 3 clinical trials.^1^, ^2^, ^3^, ^4^

Gantenerumab is a subcutaneously administered, fully human, and anti‐Aβ immunoglobulin 1 monoclonal antibody with highest affinity for aggregated Aβ, including oligomers, fibrils, and plaques. It removes Aβ through microglia‐mediated phagocytosis and promotes amyloid plaque clearance.^5^, ^6^

In prior studies, gantenerumab showed no clinical efficacy, but effects on biomarkers of amyloid pathology and neurodegeneration.^7^, ^8^ More specifically, amyloid positron emission tomography (PET) load, cerebrospinal fluid (CSF) phosphorylated tau (p‐tau) protein 181, total tau (t‐tau) protein, and neurogranin decreased after 104 weeks of treatment with 225 mg of gantenerumab every 4 weeks.

Using a four times higher dose of gantenerumab compared to these earlier trials, we conducted two phase 3 trials (GRADUATE I and II) to determine the efficacy and safety of gantenerumab in persons with early symptomatic AD.^3^

While the phase 3 trials with gantenerumab did not show significant clinical efficacy, we here report a comprehensive analysis of biomarker changes over 116 weeks of treatment with gantenerumab versus placebo that will help inform on the discrepancies between different amyloid‐removing antibodies and enhance our understanding of the utility and shortcomings of fluid biomarkers as surrogates for amyloid and tau PET, clinical efficacy, and treatment monitoring. In detail, we provide an in‐depth analysis of gantenerumab treatment effects with imaging and fluid biomarkers (CSF and blood plasma) that assess fundamental disease processes of amyloid pathology and metabolism (amyloid PET, Aβ40, Aβ42), tau pathology (tau PET, p‐tau181, p‐tau217), neurodegeneration (volumetric magnetic resonance imaging [vMRI], t‐tau, neurofilament light chain [NfL]), cerebral perfusion (dual‐phase amyloid PET), synaptic dysfunction and degeneration (neurogranin, neuronal pentraxin‐2 [NPTX2], and alpha‐synuclein [α‐syn]), glial activation and neuroinflammation (soluble triggering receptor expressed on myeloid cells 2 [sTREM2], glial fibrillary acidic protein [GFAP], S100 calcium‐binding protein B [S100B], and chitinase‐3‐like protein 1 [YKL‐40]), as well as other biomarkers broadly linked to AD (growth differentiation factor 15 [GDF15], insulin‐like growth factor‐binding protein 7 [IGFBP7]; Table S1 in supporting information).

## 2 METHODS

### 2.1 Study design

The study design was described elsewhere.^3^ Briefly, the GRADUATE I and II trials were global phase 3, multicenter, randomized, double‐blind, placebo controlled, and parallel‐group trials. In total, 1965 participants were recruited from 288 sites in 30 countries. Key eligibility criteria were 50 to 90 years of age and evidence of mild cognitive impairment due to AD or mild dementia due to AD. All enrolled participants had to be amyloid positive either by a visual read amyloid PET scan or by a CSF p‐tau181/Aβ42 ratio of > 0.024 on the Elecsys test system (Roche Diagnostics). Participants were randomly assigned in a 1:1 ratio to receive subcutaneous gantenerumab or placebo. Over a titration period of 9 months, participants reached a target dose of 510 mg gantenerumab every 2 weeks. Before every dose increase and throughout the study, participants underwent MRI monitoring to assess the presence of amyloid related imaging abnormalities (ARIAs). The primary outcome was the change from baseline in the Clinical Dementia Rating Sum of Boxes (CDR‐SB) at week 116.

### 2.2 Imaging biomarkers

#### 2.2.1 Amyloid PET to assess brain amyloid load

Amyloid PET scans were performed at screening, week 52, week 104, or week 116 in a subset of patients who had their amyloid status at screening determined by amyloid PET and who consented to participate in the amyloid PET substudy (Tables S1 and S2 in supporting information). Each parent study (GRADUATE I and II) had an individual amyloid PET substudy.^3^ [18F]florbetaben or [18F]flutemetamol were the two selected amyloid PET radioligands used according to country and site availability. The same radioligand had to be used for an individual participant throughout the study. Each participant at each visit underwent a total of 20 minutes (4 × 5 minutes of acquisition) of serial PET imaging starting 90 minutes (± 1 minute) post intravenous injection of 300 MBq (± 20%) of [18F]florbetaben or 185 MBq (± 10%) of [18F]flutemetamol. PET images were processed in PMOD (PMOD Technologies) according to the following steps: motion correction, time averaging, smoothing to reach a common resolution of 7.0 mm (in‐plane) and 8.0 mm (axial) as described in Joshi et al.,^9^ then co‐registration to a time matched T1‐weighted (T1w) MRI.

For each amyloid PET scan, a standardized uptake value ratio (SUVR) was calculated in a volume‐weighted neocortical composite region (composed of frontal, parietal, temporal, and cingulate cortex) using the whole cerebellum as the reference region. This SUVR value was then converted to a Centiloid (CL) value to enable comparability across different amyloid PET tracers.^10^ CL relations for the two tracers (using the whole cerebellum as the reference region) are: Florbetaben: CL = 175.6 x SUVR – 174.2 and Flutemetamol: CL = 143.5 x SUVR: – 141.1.

#### 2.2.2 Dual‐phase amyloid PET to assess perfusion

An additional substudy included a dual time window protocol as part of the longitudinal [18F]florbetaben amyloid PET.^3^ The first acquisition (perfusion phase) began at the moment of [18F]florbetaben injection and lasted for 30 minutes, while the second acquisition (delayed phase) started 90 minutes after injection and lasted for 20 minutes. The dual time window protocol was collected at screening, week 52, week 104, or week 116 at a subset of imaging centers in a subset of participants who consented to participate in the substudy (Table S1).

The two acquisitions (i.e., perfusion and delayed phases) were analyzed in combination to support a PET kinetic analysis: briefly, the two sequences were independently corrected for interframe subject motion using a rigid registration–based algorithm. The time‐averaged delayed sequence obtained after motion correction was rigidly aligned to a time‐matched T1w MRI scan. The time‐averaged perfusion sequence obtained after motion correction was rigidly aligned to the co‐registered time‐averaged delayed sequence. The resulting transformation matrices (delayed sequence to T1w MRI scan, and perfusion sequence to co‐registered delayed sequence) were concatenated and applied to each motion‐corrected frame of the delayed and perfusion sequence to align them to the T1w MRI scan. Finally, the two co‐registered sequences were time‐merged.

**RESEARCH IN CONTEXT**

1. **Systematic review**: Treatment effects with the anti‐amyloid antibody gantenerumab from the GRADUATE I and II phase 3 studies were reviewed in the context of imaging and fluid biomarkers of amyloid and tau pathology, neurodegeneration, and neuroinflammation.
2. **Interpretation**: While gantenerumab was not associated with clinically meaningful efficacy in early Alzheimer's disease (AD), the GRADUATE I and II trials provided evidence of robust and sustained biomarker changes associated with both amyloid and tau pathology, as well as neurodegeneration and neuroinflammation.
3. **Future directions**: Results of this study inform on the utility and shortcomings of biomarkers as indicators of amyloid removal, surrogates of clinical efficacy, monitoring biomarkers, and proof of disease modification and their use in ongoing and future clinical trials in AD patients.

Regions of interest (ROIs) were delineated on the time‐matched T1w MRI scan according to the Desikan–Killiany and the Destrieux atlases available with FreeSurfer 6.0 (see section 2.2.4 for additional details). Time activity curves (TACs) of the time‐merged PET frames were extracted for the following ROIs: cerebellar cortex (considered the reference region), frontal lobe, parietal lobe, temporal lobe, occipital lobe, meta‐temporal ROI (as defined in the section 2.2.4), and composite neocortex (frontal, cingulate, parietal, and temporal cortex).

The relative blood flow parameter R1 was estimated for each ROIs using the simplified reference tissue model (SRTM), with the cerebellar cortex as reference region.^11^

The prespecified analysis for the dual‐phase amyloid PET was based on the pooled dataset of GRADUATE I and II studies due to the small number of dual‐phase acquisitions within each study.

#### 2.2.3 Tau PET

Tau PET scans were performed at baseline, week 52, week 104, or week 116 in a subset of participants who consented to participate in the longitudinal tau PET substudy (Table S3 in supporting information).^3^ In contrast to the amyloid PET substudies, participants from both GRADUATE I and II studies were enrolled in a single tau PET substudy. Each participant at each timepoint underwent a total of 30 minutes (6 × 5 minutes) acquisition of serial PET imaging starting 60 minute (± 1 minute) post intravenous injection of 259 MBq (± 10%) of [18F]GTP1.^12^ PET images were processed in PMOD (PMOD Technologies) according to the following steps: motion correction, time averaging, smoothing to reach a common resolution of 7.0 mm (in‐plane) and 8.0 mm (axial), then co‐registration to a time‐matched T1w MRI scan.

The inferior cerebellar cortex was used as the reference region to calculate the median SUVR values in two sets of regions of interest, one based on the brain lobes: medial temporal lobe (excluding the hippocampus), lateral temporal lobe, parietal lobe, and frontal lobe; the other on regions reflecting Braak stages: Braak I and II, Braak III and IV, and Braak V and VI.^13^ The prespecified analysis for the tau PET images was based on the pooled dataset of GRADUATE I and II studies due to the small number of tau PET scans within each study.

#### 2.2.4 MRI

MRI scanners were configured following the Alzheimer's Disease Neuroimaging Initiative (ADNI) 3 recommended sequences^14^ and T1w magnetic resonance scans were collected in all participants at screening, week 48, week 104, and week 116 (Table S1).^3^ Images were analyzed according to two different approaches: a direct registration‐based volumetric difference and indirect cross‐sectional surface‐based analysis. The volumetric analysis was based on the Jacobian integration method,^15^ which uses a pair of scans (baseline and post‐baseline) to infer volumetric changes between the two visits from the non‐linear registration transform. The whole brain and whole cortex ROI were defined in the baseline image and used to calculate baseline volumes, as well as volume changes with this approach. The hippocampal volume was calculated with an unbiased patient template from all patient visits^16^ using the European Alzheimer's Disease Consortium–ADNI Harmonized Hippocampal Protocol.^17^

The surface‐based analysis was performed using the longitudinal stream of FreeSurfer 6.0.^18^, ^19^ This approach was used to calculate the change in mean cortical thickness of the whole cortex and a meta temporal ROI composed of the entorhinal, fusiform, inferior temporal, and middle temporal regions.^20^

For both approaches, values obtained after a scanner change/upgrade were excluded from further analysis. Finally, change in lateral ventricular volume was calculated based on T1w segmentation of each individual T1w image separately using a patch‐based method.^21^

### 2.3 Fluid biomarkers

#### 2.3.1 CSF biomarkers

CSF samples were collected via lumbar puncture at screening, week 104, or week 116 in a subset of participants who had amyloid status at screening determined by CSF (Table S3) between 8 am and 12 pm.^3^ A volume of 12 mL of CSF was collected in a 15 mL sterile conical screw top tube (Sarstedt 62.554.205). If the first 1 to 2 mL of CSF were colored red, the CSF was discarded and a new collection tube was used to collect more CSF. Tubes were gently inverted two to three times before centrifuging at 2000 × g at 4°C for 10 minutes. Centrifugation happened within 30 minutes of CSF sample collection. CSF (0.5 mL) was aliquoted in 0.5 mL sterile screwcap cryovials (Sarstedt 72.730.005) and immediately frozen at –70°C and shipped on dry ice to the measuring site.

Samples were thawed at room temperature and measured with commercially available Aβ42, p‐tau181, and t‐tau Elecsys immunoassays on the cobas e 601 instrument.^22^, ^23^ Aβ40, neurogranin, NfL, GFAP, sTREM2, YKL‐40, S100B, α‐syn, and NPTX2 were measured using robust prototype assays from the Elecys NeuroToolKit (Roche Diagnostics International Ltd.)^24^ on the cobas e 411 instrument at Labcorp Translational Biomarker Solutions (Indianapolis, Indiana, USA). All immunoassays used were technically validated and qualified for the purpose of this study. CSF Aβ42 was always measured as the first immunoassay per sample to avoid loss of Aβ42 by adhering to the pipette tip of the instrument due to prior pipetting from the same sample. All samples from all timepoints from a given patient were measured in the same run on the same day to avoid run‐to‐run and day‐to‐day variability. The prespecified analysis for CSF biomarkers was based on the pooled dataset of GRADUATE I and II studies due to the small number of CSF samples within each study.

#### 2.3.2 Blood‐based biomarkers

Plasma samples were collected in all patients via venipuncture at screening or baseline, week 24, week 52, week 104, and week 116 (Table S1).^3^ Two × 6 mL whole blood was collected in 6 mL potassium ethylenediaminetetraacetic acid blood collection tubes (Becton Dickinson #367863). Tubes were gently inverted 8 to 10 times before centrifuging at 1500 × g at 4°C for 10 minutes. Centrifugation happened within 60 minutes of blood sample collection at the clinical site to minimize impact of preanalytical sample handling.^25^ Plasma (0.5 mL) was aliquoted in 0.5 mL sterile screwcap cryovials (Sarstedt 72.730.005) and immediately frozen at –70°C and shipped on dry ice to the measuring site.

Samples were thawed at room temperature and measured with Aβ42, Aβ40, t‐tau, p‐tau181, p‐tau217, GFAP, NfL, sTREM2, YKL‐40, GDF15, and IGFBP7 robust prototype immunoassays from the Elecys NeuroToolKit (Roche Diagnostics International Ltd.)^24^ on the cobas e 601 instrument at Microcoat Biotechnologie GmbH (Bernried, Germany). All immunoassays used were technically validated and qualified for the purpose of this study. Aβ42 was always measured as the first immunoassay per aliquot to avoid loss of Aβ42 by adhering to the pipette tip of the instrument due to prior pipetting from the same sample. All samples from all timepoints from a given patient were measured in the same run on the same day to avoid run‐to‐run and day‐to‐day variability.

### 2.4 Statistical analysis

Biomarker values from samples of sufficient quality that were below the lower limit of quantification (LLoQ) were imputed to 0.5 x LLoQ; biomarker values from samples of sufficient quality that were above the upper limit of quantification (ULoQ) were capped at ULoQ. Biomarker data were log10 transformed.

For imaging biomarkers, a mixed model repeated measures (MMRM) was used to estimate the mean change in biomarker from baseline to week 116. The model included the change from baseline in biomarker as the dependent variable, while adjusting for treatment arm (as categorical), visit (as categorical), apolipoprotein E (*APOE*) ε4 status (as categorical; carrier vs. non‐carrier), type of tracer (for amyloid PET only, as categorical; Florbetaben vs. Flutemetamol), baseline biomarker, baseline biomarker‐by‐visit and treatment‐by‐visit interaction. Visit was treated as the repeated variable within a participant. An unstructured variance–covariance matrix was applied to model the within‐participant errors; in the case of non‐convergence, compound symmetry was used together with a robust “sandwich” estimator of standard error.

For each CSF biomarker, log10 transformation was applied to the data before statistical analysis. An analysis of covariance (ANCOVA) model was built using the change from baseline to week 116 as dependent variable and treatment arm (as categorical), *APOE* ε4 status (as categorical; carrier vs. non‐carrier), and baseline biomarker as covariates. The summary statistics and treatment effects were estimated after the appropriate back‐transformation of the original model parameters to obtain the geometric means, geometric means ratio, and the % difference in geometric mean (relative to placebo) at week 116 for each treatment arm.

For each plasma biomarker, log10 transformation was applied to the data before statistical analysis. An MMRM model was constructed using the change from baseline as dependent variable and the following covariates: treatment arm (as categorical), visit (as categorical), treatment‐by‐visit interaction, *APOE* ε4 status (as categorical; carrier vs. non‐carrier), and baseline biomarker and baseline biomarker‐by‐visit. Visit was treated as the repeated variable within a participant and an unstructured variance–covariance matrix was applied to model the within‐participant errors. In case of non‐convergence, compound symmetry was used, together with a robust “sandwich” estimator of standard error.

No multiplicity adjustment of *P* values was performed for any of the biomarkers. All statistical analyses were performed using R v4.0.4.

## 3 RESULTS

### 3.1 Amyloid PET

**FIGURE 1:**
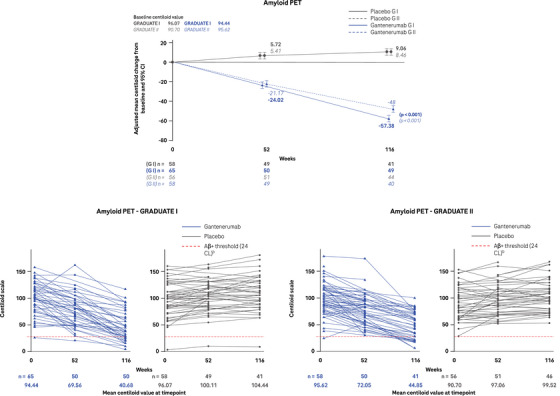
Amyloid PET. Top row shows the adjusted mean change from baseline to week 116 in the amyloid load as measured by Centiloid using amyloid PET imaging (G I solid line and G II dotted line). Bottom row shows the individual trajectory of amyloid load for each participant enrolled in the amyloid PET substudy of G I (left) and G II (right). Aβ, amyloid beta; CI, confidence interval; CL, Centiloid; G I, GRADUATE I; G II, GRADUATE II; PET, positron emission tomography.

Baseline characteristics were well balanced between participants treated with gantenerumab and placebo across both amyloid PET substudies (Table S2). At week 116, adjusted mean amyloid load by PET were lower in gantenerumab versus placebo treated participants (Figure 1). The difference in the adjusted mean change from baseline to week 116 in amyloid load between the gantenerumab group and the placebo group was –66.44 CL (95% confidence interval [CI]: –74.71 to –58.16) in GRADUATE I and –56.46 CL (95% CI: –64.36 to –48.56) in GRADUATE II.

In GRADUATE I, the mean (± standard deviation [SD]) amyloid load at screening was at 94.44 ± 26.48 and 96.07 ± 31.47 CL and reached 40.68 ± 27.39 and 104.44 ± 33.15 CL at week 116 in the gantenerumab and placebo groups, respectively (Figure 1). In GRADUATE II, the mean (± SD) amyloid load at screening was at 95.62 ± 30.76 and 90.7 ± 30.80 CL and reached 44.85 ± 26.67 and 99.52 ± 27.72 CL at week 116 in the gantenerumab and placebo groups, respectively (Figure 1). At week 116, an amyloid‐negative status (≤ 24 CL) was attained in 28.0% and 2.4% of the participants receiving gantenerumab and placebo, respectively, in GRADUATE I and in 26.8% and 0% of the participants receiving gantenerumab and placebo, respectively, in GRADUATE II (Figure 1).

### 3.2 Dual‐phase amyloid PET (perfusion)

Baseline R1 values were similar in the gantenerumab and placebo groups in all regions considered, with the highest values found in the occipital lobe (mean ± SD of 0.97 ± 0.07 and 0.98 ± 0.07 in the gantenerumab and placebo groups, respectively), and the lowest values found in the meta‐temporal ROI (mean ± SD of 0.81 ± 0.07 and 0.81 ± 0.07 in the gantenerumab and placebo groups, respectively). At week 116, a decrease of R1 was observed in both groups treated with gantenerumab or placebo in all six regions considered, with the greatest decrease in the meta‐temporal ROI (adjusted mean change from baseline ± standard error [SE] of –0.043 ± 0.007 and –0.042 ± 0.008 in the gantenerumab and placebo groups, respectively; Figure S1 in supporting information). No treatment effect was observed at week 116 in any of the regions considered.

### 3.3 Tau PET

Baseline characteristics were well balanced between participants treated with gantenerumab and placebo in the tau PET substudy (Table S3). Tau PET SUVR values at baseline were similar in the gantenerumab and placebo groups in all regions considered and followed the typical pattern of AD: When considering the lobar parcellation of the brain, higher SUVR values at baseline were observed in the medial temporal lobe (SUVR = 1.47 ± 0.23 and SUVR = 1.50 ± 0.25 in the gantenerumab and placebo groups, respectively), followed by the lateral temporal (SUVR = 1.46 ± 0.35 and SUVR = 1.44 ± 0.31 in the gantenerumab and placebo groups, respectively), parietal (SUVR = 1.30 ± 0.35 and SUVR = 1.26 ± 0.29 in the gantenerumab and placebo groups, respectively), and frontal lobes (SUVR = 1.18 ± 0.25 and SUVR = 1.16 ± 0.21 in the gantenerumab and placebo groups, respectively); when considering the Braak staging system, higher SUVR values at baseline were observed in the Braak I and II region (SUVR = 1.44 ± 0.20 and SUVR = 1.48 ± 0.20 in the gantenerumab and placebo groups, respectively), followed by Braak III and IV (SUVR = 1.39 ± 0.27 and SUVR = 1.37 ± 0.24 in the gantenerumab and placebo groups, respectively), and Braak V and VI regions (SUVR = 1.23 ± 0.26 and SUVR = 1.21 ± 0.21 in the gantenerumab and placebo groups, respectively).

**FIGURE 2:**
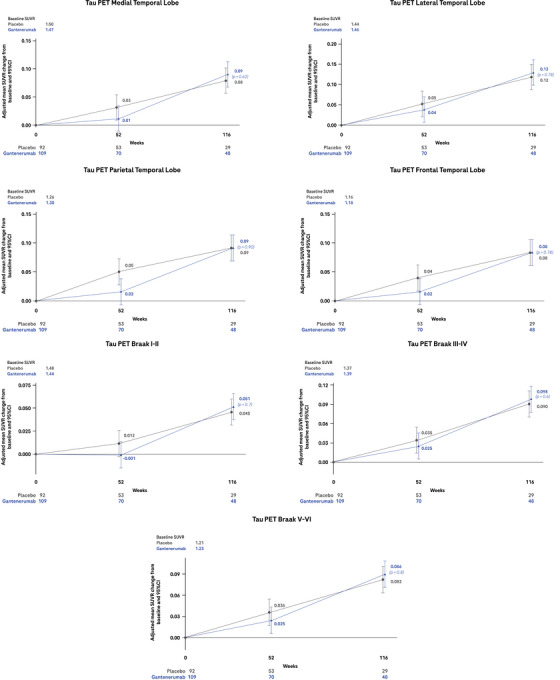
Tau PET. Adjusted mean change from baseline to week 116 in the tau load in seven brain regions of interest as measured by [18F]GTP1 tau PET SUVR. *P* values not corrected for multiplicity. CI, confidence interval; GTP1, Genentech tau probe 1; PET, positron emission tomography; SUVR, standardized uptake value ratio.

Continuous accumulation of tau was observed in both arms in all the regions considered, with the greatest increase found in the lateral temporal lobe (adjusted mean change from baseline ± SE of 0.13 ± 0.014 SUVR and 0.12 ± 0.018 SUVR in the gantenerumab and placebo groups, respectively; Figure 2).

No treatment effect was observed at week 116 in any of the regions considered.

### 3.4 vMRI

**FIGURE 3:**
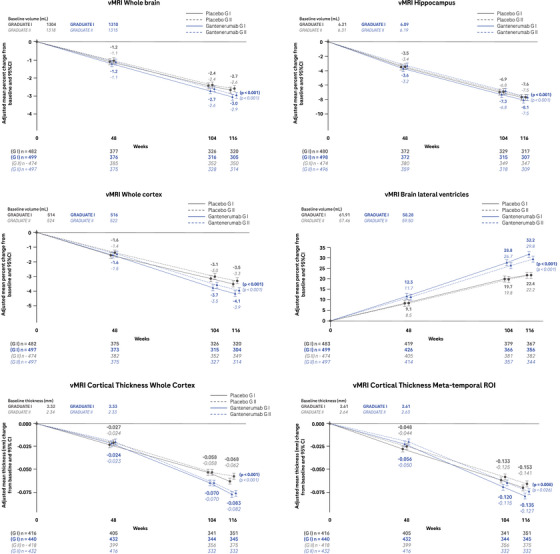
vMRI. Adjusted mean change from baseline to week 116 in vMRI in whole brain, hippocampus, whole cortex, and brain lateral ventricles, as well as the adjusted mean change from baseline to week 116 in cortical thickness of the whole cortex and temporal‐meta ROI. *P* values not corrected for multiplicity. CI, confidence interval; G I, GRADUATE I; G II, GRADUATE II; ROI, region of interest; vMRI, volumetric magnetic resonance imaging.

The volumetric pipeline showed that at week 116, participants treated with gantenerumab had a greater decrease in whole brain and whole cortical volume and a greater increase in lateral ventricular volume compared to placebo. In the whole brain region, the difference in the adjusted mean change from baseline to week 116 between the gantenerumab and placebo group was –0.32% (95% CI: –0.50 to –0.14) in GRADUATE I and –0.36% (95% CI: –0.54 to –0.18) in GRADUATE II. In the whole cortex, the difference in the adjusted mean change from baseline to week 116 between the gantenerumab and placebo group was –0.64% (95% CI: –0.87 to –0.42) in GRADUATE I and –0.64% (95% CI: –0.87 to –0.41) in GRADUATE II. In the lateral ventricular region, the difference in the adjusted mean change from baseline to week 116 between the gantenerumab and placebo group was 9.81% (95% CI: 7.92 to 11.7) in GRADUATE I and 7.62% (95% CI: 5.64 to 9.6) in GRADUATE II. Continuous volume loss was also observed in the hippocampus in both treatment groups, but with no treatment effect at week 116 (Figure 3).

The surface‐based pipeline showed that at week 116, participants treated with gantenerumab had a greater decrease in the whole cortex and in meta‐temporal ROI cortical thickness compared to placebo (Figure 3). The difference in the adjusted mean change from baseline to week 116 in the whole brain cortical thickness between the gantenerumab and placebo group was –0.016 mm (95% CI: –0.025 to –0.007) in GRADUATE I and –0.02  mm (95% CI: –0.029 to –0.011) in GRADUATE II. The difference in the adjusted mean change from baseline to week 116 in the meta‐temporal ROI between the gantenerumab and placebo group was –0.018  mm (95% CI: –0.031 to –0.005) in GRADUATE I and –0.014  mm (95% CI: –0.027 to –0.002) in GRADUATE II.

### 3.5 CSF biomarkers

**FIGURE 4:**
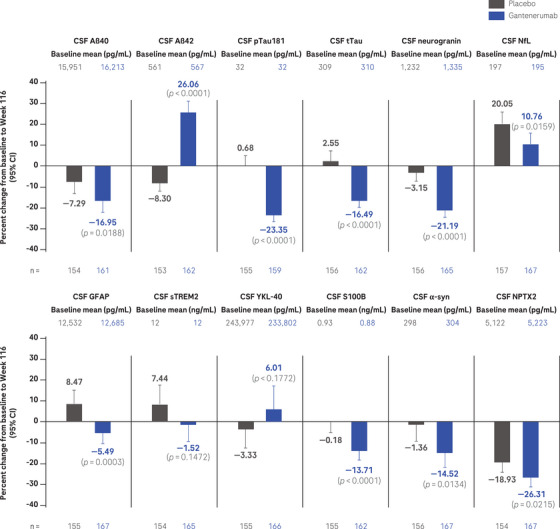
CSF biomarkers. Adjusted mean plot of ANCOVA for CSF biomarker percent change from baseline to Week 116. *P* values not corrected for multiplicity. ANCOVA, analysis of covariance; Aβ, amyloid beta; α‐syn, alpha‐synuclein; CI, confidence interval; CSF, cerebrospinal fluid; GFAP, glial fibrillary acidic protein; NfL, neurofilament light; NPTX2, neuronal pentraxin‐2; pTau, phosphorylated tau; sTREM2, soluble triggering receptor expressed on myeloid cells‐2; S100B, S100 calcium‐binding protein B; tTau, total tau; YKL‐40, chitinase 3‐like 1.

Baseline characteristics were well balanced between participants treated with gantenerumab and placebo in the subgroup of participants that provided longitudinal CSF (Table S3). At week 116, geometric mean concentrations of CSF Aβ40 (–16.95% [95% CI: –22.10 to –11.80] vs. –7.29% [95% CI: –13.17 to –1.41]), t‐tau (–16.49% [95% CI: –19.62 to –13.36] vs. 2.55% [95% CI: –2.15 to 7.25]), p‐tau181 (–23.35% [95% CI: –26.37 to –20.33] vs. 0.68% [95% CI: –3.50 to 4.86]), neurogranin (–21.19% [95% CI: –24.35 to –18.03] vs. –3.15% [95% CI: –7.14 to 0.84]), GFAP (–5.49% [95% CI: –10.20 to –0.78] vs. 8.47% [95% CI: 2.53 to 14.41]), S100B (–13.71% [95% CI: –17.89 to –9.53] vs. –0.18% [95% CI: –5.12 to –4.76]), α‐syn (–14.52% [95% CI: –20.99 to –8.05] vs. –1.36% [95% CI: –9.08 to 6.36]), and NPTX2 (–26.31% [95% CI: –30.34 to –22.28] vs. –18.93% [95% CI: –23.54 to –14.32]) were decreased in gantenerumab compared to placebo treated participants while geometric mean concentrations of Aβ42 increased (26.06% [95% CI: 21.01 to 31.11] vs. –8.30% [95% CI: –11.94 to –4.66]; Figure 4).

CSF NfL geometric mean concentrations at week 116 showed an increase in both the gantenerumab and placebo arms (10.76% [95% CI: 5.61 to 15.91] vs. 20.05% [95% CI: 14.30 to 25.80]) but the increase was less pronounced with gantenerumab versus placebo (Figure 4). There was no difference in geometric mean concentrations of CSF sTREM2 and YKL‐40 in gantenerumab compared to placebo at week 116 (Figure 4).

### 3.6 Blood‐based biomarkers

At week 116, geometric mean concentrations of plasma Aβ40 and Aβ42 were elevated with gantenerumab versus placebo, with the percent change being larger for Aβ42 (65.1% [95% CI: 61.2 to 69.3] vs. 3.2% [95% CI: 0.7 to 5.7] and 54.9% [95% CI: 51.1 to 58.7] vs. 2.0% [95% CI: –0.4 to 4.5] in GRADUATE I and II, respectively) than for Aβ40 (35.8% [95% CI: 33.3 to 38.4] vs. 2.6% [95% CI: 0.7 to 4.5] and 30.0% [95% CI: 27.4 to 32.6] vs. –0.2% [95% CI: –2.2 to 1.7] in GRADUATE I and II, respectively), and thus the Aβ42:Aβ40 ratio also increased (21.1% [95% CI: 19.8 to 22.5] vs. 1.7% [95% CI: 0.6 to 2.8] and 19.0% [95% CI: 17.9 to 20.1] vs. 1.9% [95% CI: 1.0 to 2.8] in GRADUATE I and II, respectively; Figure 5). Over 116 weeks, t‐tau, p‐tau181, p‐tau217, GFAP, NfL, sTREM2, YKL‐40, GDF15, and IGFBP7 increased in the placebo arm. Under 116 weeks of treatment with gantenerumab, the geometric mean concentrations for t‐tau (0.2% [95% CI: –2.5 to 2.9] vs. 5.6% [95% CI: 2.8 to 8.4] and –1.7% [95% CI: –4.1 to 0.7] vs. 3.9% [95% CI: 1.4 to 6.4] in GRADUATE I and II, respectively), and even more pronounced for p‐tau181 (–17.2% [95% CI: –19.2 to –15.3] vs. 8.8% [95% CI: 6.3 to 11.4] and –15.3% [95% CI: –17.2 to –13.3] vs. 7.2% [95% CI: 4.8 to 9.6] in GRADUATE I and II, respectively), p‐tau217 (–32.7% [95% CI: –35.4 to –30.3] vs. 13.2% [95% CI: 9.0 to 17.6] and –32.9% [95% CI: –35.3 to –30.0] vs. 11.8% [95% CI: 7.6 to 16.0] in GRADUATE I and II, respectively), and GFAP (–10.8% [95% CI: –13.1 to –8.5] vs. 13.5% [95% CI: 10.6 to 16.4] and –8.9% [95% CI: –11.0 to –6.8] vs. 11.8% [95% CI: 9.3 to 14.4] in GRADUATE I and II, respectively) were decreased compared to placebo (Figure 5). For NfL, sTREM2, YKL‐40, GDF15, and IGFBP7 there were no treatment effects detected between gantenerumab and placebo (Figure 5).

FIGURE 5Blood‐based biomarkers. Adjusted mean plot of plasma biomarker percent change from baseline to Week 116. An MMRM model was used on log‐transformed data. *P* values not corrected for multiplicity. Aβ, amyloid beta; CI, confidence interval; G, GRADUATE; GDF15, growth differentiation factor 15; GFAP, glial fibrillary acidic protein; IGFBP7, insulin‐like growth factor‐binding protein 7; MMRM, mixed models for repeated measures; NfL, neurofilament light; pTau, phosphorylated tau; sTREM2, soluble triggering receptor expressed on myeloid cells‐2; tTau, total tau; YKL‐40, chitinase 3‐like 1.

**Figure:**
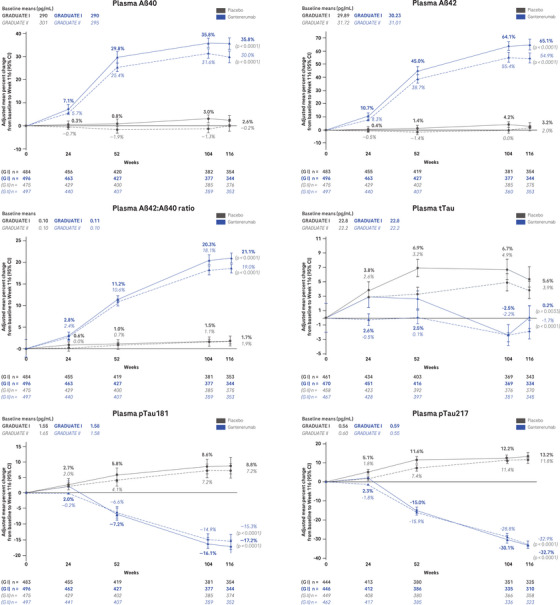

**Figure:**
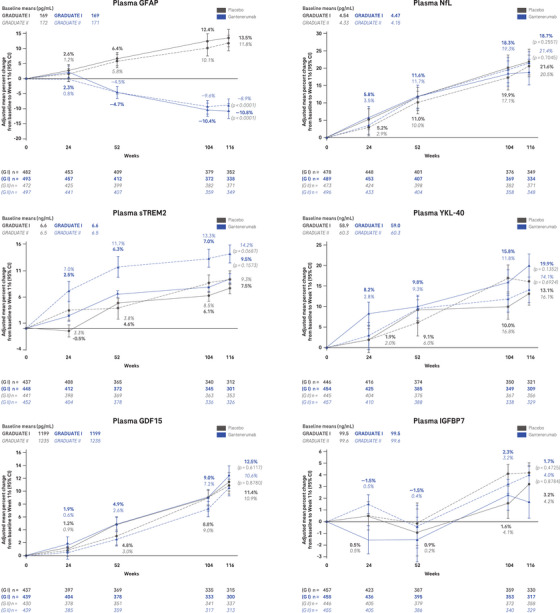

## 4 DISCUSSION

Here we report a comprehensive, prespecified analysis of imaging and fluid biomarker effects of treatment with gantenerumab versus placebo over 116 weeks on fundamental disease processes of amyloid and tau pathology, neurodegeneration, and neuroinflammation.

Gantenerumab significantly reduced amyloid plaque load as measured by amyloid PET. Twenty‐eight percent of participants in GRADUATE I and 27% in GRADUATE II were below the amyloid positivity threshold of 24 CL at week 116. The magnitude of decrease in amyloid load with gantenerumab after 27 months of treatment was comparable^1^, ^2^ or lower^4^ than the decrease reported for other amyloid‐removing antibodies at 18 months. However, a direct comparison is difficult due to differences in dose titration periods across those studies ranging from 0 to 9 months.

The observed increase in plasma Aβ40 and Aβ42 species in the GRADUATE I and II studies can probably be attributed to the effect that in the blood, large quantities of gantenerumab bind the much lower abundant Aβ40 and Aβ42 species and thereby prolong their half‐life, which leads to an overall increase in measured plasma Aβ40 and Aβ42 levels. In untreated individuals, Aβ40 and Aβ42 are degraded by insulin‐degrading enzyme (IDE) from blood cells (leukocytes and erythrocytes) at positions 19 and 20, which are bound by certain therapeutic antibodies.^26^ This effect seems to be in line with previous reports from other anti‐amyloid antibodies like lecanemab^2^ but at a much lower magnitude of increase compared to non‐amyloid plaque–removing antibodies like crenezumab^27^ and solanezumab.^28^

In CSF, in which much smaller quantities of gantenerumab and much larger amounts of Aβ40 and Aβ42 are present, the changes in Aβ40 and Aβ42 levels after 116 weeks of treatment with gantenerumab versus placebo differ from the situation in plasma. In CSF, treatment with gantenerumab decreased Aβ40 while increasing Aβ42, which supports the notion that the increase in CSF Aβ42 is likely not due to a pronged half‐life of binding to gantenerumab like in plasma but could rather be a direct reflection of clearance of Aβ42 from aggregated amyloid in the brain. This effect seems to be in line with previous reports from other amyloid plaque–removing antibodies like lecanemab^2^ but is different compared to non‐amyloid plaque–removing anti‐amyloid antibodies like crenezumab^27^ and solanezumab^28^ in which also in CSF both Aβ42 and Aβ40 were markedly elevated under treatment. This difference could be attributed to the higher binding affinity of crenezumab and solanezumab to soluble Aβ species.^27^, ^28^

When assessing the biomarker effects of gantenerumab on tau biomarkers, tau PET showed continuous tau accumulation without a treatment effect. One limitation of this study is the small sample size of the tau PET substudy, which might have prevented the detection of small treatment effects with gantenerumab. However, when looking at tau PET data across amyloid plaque–removing antibodies there is no consistent pattern between treatment effects on tau PET and clinical efficacy.^1^, ^2^, ^3^, ^4^

Fluid biomarkers of tau showed a consistent decrease under treatment with gantenerumab compared to placebo across CSF and plasma as well as across different species (t‐tau, p‐tau181, and p‐tau217). These observations are in direction and magnitude comparable to changes seen with other amyloid plaque–removing antibodies.^1^, ^2^, ^4^ Given the lack of treatment effects on tau pathology as measured by tau PET for some amyloid plaque–removing antibodies (e.g., donanemab,^4^ gantenerumab^3^), the robust and consistent treatment effects on fluid biomarkers of tau might be more reflections of amyloid plaque removal, resulting in a lowering of secretion of p‐tau species from neurons, rather than a direct treatment effect on tau tangle pathology. An alternative explanation is that fluid biomarkers of tau allow a more sensitive measurement of treatment effects than tau PET imaging.

Under treatment with gantenerumab versus placebo, volumetric MRI showed a slightly greater reduction in whole brain and whole cortical volume, with concurrent greater lateral ventricular expansion while the hippocampal volume was not differentially affected. Further surface‐based analysis echoed these results, showing slightly increased cortical thinning of the whole cortex and meta‐temporal ROI under treatment with gantenerumab compared to placebo. This finding is in line with several other amyloid plaque–removing antibodies^1^, ^2^, ^4^ but its interpretation is still unclear. The fact that clinical findings and other biomarkers related to neurodegeneration (i.e., NfL) point toward a beneficial effect of anti‐amyloid antibodies suggests that the reported volume loss might not represent accelerated neurodegeneration.^29^ Other factors such as treatment‐induced resolution of inflammation or CSF fluid shifts have been suggested to cause these brain volume changes.^30^

From dual‐phase amyloid PET (perfusion) imaging, the tracer delivery rate R1, a measure correlated with cerebral blood flow^31^ and energy metabolism,^32^ had the potential to elucidate this phenomenon by providing information on the functional and metabolic state of the cortical tissue. However, while we were able to detect decreases in R1 in both gantenerumab and placebo groups in all the brain regions considered, we did not detect any treatment effect. One possible explanation could be the limited sample size of this substudy.

For NfL as a fluid biomarker of neurodegeneration we detected a reduced accumulation in CSF under treatment with gantenerumab compared to placebo but no treatment effect in plasma. The lack of a robust treatment effect in plasma is consistent across amyloid‐targeting antibodies.^2^, ^4^, ^27^ However, comparing changes in NfL after treatment to gantenerumab^3^ and lecanemab^2^ only gantenerumab shows a treatment effect in the form of a reduced accumulation versus placebo. A possible explanation could be differences in analytical sensitivity and precision of the different immunoassays used. At any rate, the data seems to suggest that NfL is so far not a robust biomarker to indicate clinical efficacy with amyloid‐targeting antibodies in AD.

CSF neurogranin is a synaptic biomarker that is increased in untreated AD patients^33^ and was found to decrease under treatment with gantenerumab. This finding is in line with previous gantenerumab studies^8^ and with results from other amyloid‐targeting antibodies.^2^, ^27^ NPTX2 as another synaptic biomarker that shows decreased levels in CSF in untreated AD patients^34^ decreased even further under treatment with gantenerumab versus placebo. The reasons and implications are unclear at this stage.

Biomarkers of microglia and astroglia activation showed inconsistent results. Only GFAP was decreased in CSF and plasma under treatment with gantenerumab compared to placebo. Increases in plasma GFAP have been closely linked to amyloid pathology in AD^35^ so the treatment effect seen with gantenerumab and other amyloid plaque–removing antibodies suggests this to reflect amyloid removal.^2^, ^4^

We also evaluated GDF15 and IGFBP7 as potentially meaningful biomarkers associated with AD. While we did not detect any treatment effects with gantenerumab, GDF15 showed a robust and consistent increase over the course of the studies, which supports a potential role as a biomarker of disease progression in AD.^36^

In conclusion, robust treatment effects with gantenerumab compared to placebo were observed across several biomarkers associated with amyloid and tau pathology with a more mixed picture for biomarkers of neurodegeneration and neuroinflammation. Comparing these biomarker changes to treatment effects reported for other amyloid plaque–removing antibodies^1^, ^2^, ^4^ the findings suggest that (1) p‐tau and GFAP have utility as indicators of amyloid plaque removal; (2) NfL is not a reliable indicator of clinical efficacy in plasma or CSF; (3) vMRI might be confounded by non‐neurodegenerative brain volume changes, which probably renders it unsuitable as a robust biomarker reflecting clinical efficacy; and (4) treatment effects on tau PET are inconsistent across amyloid plaque–removing antibodies and therefore likely not a robust indicator of clinical efficacy.

Limitations of the study are the limited sample size for PET and CSF subpopulations and the generalizability of those findings to the overall study population, the lack of a specific collection time for blood samples, the lack of clinical efficacy of gantenerumab, and the lack of type I error control in this investigative setting. In addition, because only a subset of study participants had their amyloid removed to below the amyloid negativity threshold, the effects of full clearance of amyloid plaques on biomarkers cannot be analyzed in this study.

Further comparisons of absolute biomarker levels at the end of treatment with levels of age‐matched non‐AD controls will help us understand the magnitude of achieved “normalization” of biomarker levels across AD therapies. This will further improve our understanding of the utility of fluid biomarkers as surrogates for amyloid and tau PET, clinical efficacy, and treatment monitoring.

## CONFLICT OF INTEREST STATEMENT

Tobias Bittner is a full‐time employee of F. Hoffmann‐La Roche Ltd and Genentech, Inc., a member of the Roche Group, and owns stock in F. Hoffmann‐La Roche Ltd. Matteo Tonietto, Gregory Klein, Anton Belousov, Vittorio Illiano, Paul Delmar, and Susanna Gobbi are full‐time employees of F. Hoffmann‐La Roche Ltd and own stock in F. Hoffmann‐La Roche Ltd. At the time of the study, Christopher Galli was full‐time employee of F. Hoffmann‐La Roche Ltd and owned stock in F. Hoffmann‐La Roche Ltd. Marzia A. Scelsi is a full‐time employee of Roche Products Ltd. Nicola Voyle is a full‐time employee of Roche Products Ltd. and owns stock in F. Hoffmann‐La Roche Ltd. Muhamed Barakovic is an employee of Hays plc and a consultant for F. Hoffmann‐La Roche Ltd. Maryam Abaei, Erica Silvestri, and Antonio Napolitano are employees of A4P Consulting Ltd. and consultants for F. Hoffmann‐La Roche Ltd. Kaj Blennow has served as a consultant and on advisory boards for AC Immune, Acumen, ALZPath, AriBio, BioArctic, Biogen, Eisai, Lilly, Moleac Pte. Ltd., Novartis, Ono Pharma, Prothena, Roche Diagnostics, and Siemens Healthineers; has served on data monitoring committees for Julius Clinical and Novartis; has given lectures, produced educational materials, and participated in educational programs for AC Immune, Biogen, Celdara Medical, Eisai, and Roche Diagnostics; and is a co‐founder of Brain Biomarker Solutions in Gothenburg AB (BBS), which is a part of the GU Ventures Incubator Program, outside the work presented in this paper. Frederik Barkhof has served on steering committees or as data safety monitoring board member for Biogen, Merck, Eisai, and Prothena; as an advisory board member for Combinostics, Scottish Brain Sciences; and as a consultant for Roche, Celltrion, Rewind Therapeutics, Merck, and Bracco. He has research agreements with ADDI, Merck, Biogen, GE Healthcare, and Roche and is a co‐founder and shareholder of Queen Square Analytics Ltd. Author disclosures are available in the supporting information.

## CONSENT STATEMENT

All human subjects that participated in this study provided informed consent.

## Supporting information



Supporting Information

Supporting Information

Supporting Information

Supporting Information

Supporting Information
