# Supplementary material for: Biomarker treatment effects in two phase 3 trials of gantenerumab
Source: Alzheimers Dement. 2025 Jan 30;21(2):e14414. doi: 10.1002/alz.14414 (PMC11848197; doi:10.1002/alz.14414)
Supplement: Supplementary file 1 — Supporting Information [file ALZ-21-e14414-s001.docx]

**Supplementary Figure 1. Change from Baseline in Amyloid Levels on Perfusion PET**


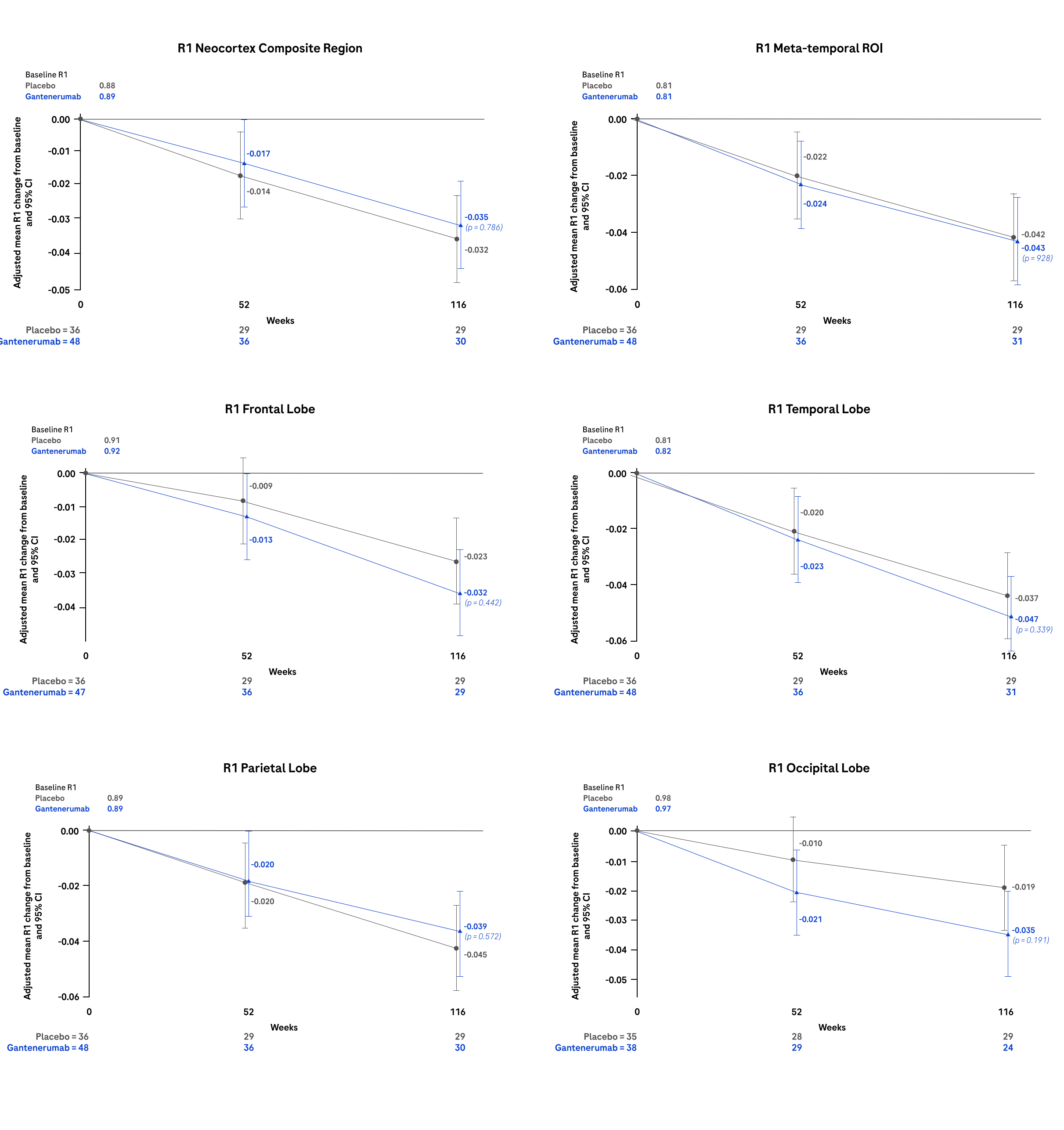


Adjusted mean change from baseline to week 116 in the relative tracer delivery R1 in 6 brain regions of interest as measured by dual-phase amyloid PET. p values not corrected for multiplicity. CI, confidence interval; PET, positron emission tomography.
