# Supplementary material for: Biomarker treatment effects in two phase 3 trials of gantenerumab
Source: Alzheimers Dement. 2025 Jan 30;21(2):e14414. doi: 10.1002/alz.14414 (PMC11848197; doi:10.1002/alz.14414)
Supplement: Supplementary file 2 — Supporting Information [file ALZ-21-e14414-s003.docx]

**Supplementary Table 1. Overview of biomarker methods and schedule of assessments.**

| **Biomarker** | **Methodology used** | **Schedule of assessments** |
| --- | --- | --- |
| Amyloid PET | [18F]Florbetaben and [18F]Flutemetamol with SUVR and CL  neocortical composite region (composed of frontal, parietal, temporal, and cingulate cortex)  using whole cerebellum as reference region | SCR/BL, week 52, 104 or 116  in a subset of participants within two separate substudies for GRADUATE I and II |
| Dual-phase amyloid PET | [18F]Florbetaben with R1 in  frontal lobe  parietal lobe  temporal lobe  occipital lobe  meta-temporal ROI  composite neocortex  using cerebellar grey matter as reference region | SCR/BL, week 52, 104 or 116  in a subset of participants within one substudy across GRADUATE I and II |
| Tau PET | [18F]GTP1 in SUVR in  medial temporal lobe (excluding the hippocampus)  lateral temporal lobe  parietal lobe  frontal lobe  Braak I-II  Braak III-IV  Braak V-VI  using the inferior cerebellar grey matter as reference region | BL, week 52, 104 or 116  in a subset of participants within one substudy across GRADUATE I and II |
| vMRI | Volume change in  whole brain  bilateral hippocampus  whole cortex  brain lateral ventricles  Cortical thickness in  whole cortex  temporal-meta ROI | SCR/BL, week 48, 104, 116  in all participants |
| CSF | Roche Elecsys Neurotoolkit  Aβ40  Aβ42  tTau  pTau181  neurogranin  NfL  GFAP  sTREM2  YKL-40  S100B  alpha-synuclein  NPTX2 | SCR, week 52, 104 or 116  in a subset of participants |
| Plasma | Roche Elecsys Neurotoolkit  Aβ40  Aβ42  Aβ42:Aβ40 ratio  tTau  pTau181  pTau217  GFAP  NfL  sTREM2  YKL-40  GDF15  IGFBP7 | SCR/BL, week 24, 52, 104, 116  in all participants |

Aβ amyloid-beta, BL baseline, CL centiloids, CSF cerebrospinal fluid, GDF15 growth differentiation factor 15, GFAP glial fibrillary acidic protein, GTP1 Genentech Tau Probe 1, IGFBP7 Insulin-like growth factor-binding protein 7, NfL neurofilament light chain, NPTX2 neuronal pentraxin-2, PET positron emission tomography, pTau phosphorylated tau, ROI region of interest, S100B calcium-binding protein B, SCR screening, sTREM2 soluble Triggering Receptor Expressed On Myeloid Cells 2, SUVR standard uptake value ratio, tTau total tau, vMRI volumetric magnetic resonance imaging, YKL-40 chitinase-3-like protein 1.
