# Supplementary material for: Biomarker treatment effects in two phase 3 trials of gantenerumab
Source: Alzheimers Dement. 2025 Jan 30;21(2):e14414. doi: 10.1002/alz.14414 (PMC11848197; doi:10.1002/alz.14414)
Supplement: Supplementary file 3 — Supporting Information [file ALZ-21-e14414-s002.docx]

**Supplementary Table 2. Baseline Characteristics of the amyloid PET longitudinal substudy population**

| **Demographics and clinical characteristics** | **GRADUATE I  Amyloid PET substudy**  **(N = 123)** | | **GRADUATE I**  **(N = 984)** | | **GRADUATE II  Amyloid PET substudy**  **(N = 114)** | | **GRADUATE II**  **(N = 975)** | |
| --- | --- | --- | --- | --- | --- | --- | --- | --- |
|  | **Placebo  (n = 58)** | **Gantenerumab  (n = 65)** | **Placebo**  **(n = 485)** | **Gantenerumab  (n = 499)** | **Placebo  (n = 56)** | **Gantenerumab  (n = 58)** | **Placebo  (n = 477)** | **Gantenerumab  (n = 498)** |
| **Age, mean (SD)** | 73.2 (8.6) | 72.2 (8.5) | 72.1 (7.8) | 71.1 (7.9) | 71.1 (7.5) | 70.4 (8.1) | 71.8 (7.4) | 71.6 (7.8) |
| **Sex, female, n (%)** | 29 (50.0) | 33 (50.8) | 255 (52.6) | 290 (58.1) | 27 (48.2) | 28 (48.3) | 285 (59.7) | 288 (57.8) |
| **Region, n (%)**  Western Europe and Australia  North America  Other | 27 (46.6)  24 (41.4)  7 (12.1) | 31 (47.7)  28 (43.1)  6 (9.2) | 227 (46.8)  126 (26.0)  132 (27.2) | 228 (45.7)  140 (28.1)  131 (26.3) | 27 (48.2)  14 (25.0)  15 (26.8) | 27 (46.6)  19 (32.8)  12 (20.7) | 158 (33.1)  113 (23.7)  206 (43.2) | 165 (33.1)  122 (24.5)  211 (42.4) |
| **Race, n (%)**  Asian  White  Other/unknown | 5 (8.6)  50 (86.2)  3 (5.2) | 6 (9.2)  59 (90.8)  0 (0.0) | 53 (10.9)  398 (82.1)  34 (7.0) | 52 (10.4)  414 (83.0)  33 (6.6) | 13 (23.2)  43 (76.8)  0 (0.0) | 11 (19.0)  45 (77.6)  2 (3.4) | 75 (15.7)  385 (80.7)  17 (3.6) | 56 (11.2)  424 (85.1)  18 (3.6) |
| **Ethnic group, n (%)**  Hispanic or Latino  Not Hispanic or Latino  Not stated/unknown | 9 (15.5)  48 (82.8)  1 (1.7) | 9 (13.8)  56 (86.2)  0 (0.0) | 58 (12.0)  422 (87.0)  5 (1.0) | 52 (10.4)  439 (88.0)  8 (1.6) | 5 (8.9)  51 (91.1)  0 (0.0) | 2 (3.4)  56 (96.6)  0 (0.0) | 119 (24.9)  358 (75.1)  0 (0.0) | 112 (22.5)  386 (77.5)  0 (0.0) |
| **Years of education, mean (SD)** | 13.6 (4.0) | 12.8 (3.8) | 13.6 (3.8) | 13.3 (3.7) | 15.1 (4.0) | 14.0 (4.5) | 13.3 (4.4) | 13.3 (4.2) |
| ***APOE ε4* allele, n (%)**  *0 ɛ4*  *1 ɛ4*  *2 ɛ4* | 16 (27.6)  33 (56.9)  9 (15.5) | 23 (35.4)  34 (52.3)  8 (12.3) | 157 (32.4)  241 (49.7)  87 (17.9) | 173 (34.7)  235 (47.1)  91 (18.2) | 15 (26.8)  32 (57.1)  9 (16.1) | 13 (22.4)  32 (55.2)  13 (22.4) | 156 (32.7)  254 (53.2)  67 (14.0) | 165 (33.1)  242 (48.6)  91 (18.3) |
| **Diagnosis at baseline, n (%)**  MCI due to AD  Mild AD dementia | 28 (48.3)  30 (51.7) | 35 (53.8)  30 (46.2) | 263 (54.2)  222 (45.8) | 275 (55.1)  224 (44.9) | 35 (62.5)  21 (37.5) | 38 (65.5)  20 (34.5) | 266 (55.8)  211 (44.2) | 269 (54.0)  229 (46.0) |
| **CDR-SB, mean (SD)** | 3.5 (1.6) | 3.8 (1.7) | 3.7 (1.6) | 3.7 (1.7) | 3.4 (1.4) | 3.5 (1.3) | 3.5 (1.5) | 3.7 (1.6) |
| **MMSE, mean (SD)** | 23.1 (3.2) | 23.4 (3.6) | 23.6 (3.0) | 23.5 (3.3) | 24.2 (3.3) | 23.8 (2.9) | 23.8 (3.2) | 23.6 (3.1) |

APOE apolipoprotein E, PET positron emission tomography, SD standard deviation.
