# Supplementary material for: Biomarker treatment effects in two phase 3 trials of gantenerumab
Source: Alzheimers Dement. 2025 Jan 30;21(2):e14414. doi: 10.1002/alz.14414 (PMC11848197; doi:10.1002/alz.14414)
Supplement: Supplementary file 4 — Supporting Information [file ALZ-21-e14414-s005.docx]

**Supplementary Table 3. Baseline Characteristics of the tau PET longitudinal substudy and CSF subgroup populations**

| **Demographics and clinical characteristics** | **Tau PET substudy  (N = 153)** | | **CSF subgroup**  **(N = 562)** | | **All participants**  **(N = 1,959)** |
| --- | --- | --- | --- | --- | --- |
|  | **Placebo**  **(n = 67)** | **Gantenerumab  (n = 86)** | **Placebo**  **(n = 269)** | **Gantenerumab  (n = 293)** |  |
| **Age, mean (SD)** | 72.1 (8.1) | 70.9 (8.8) | 73.1 (7.6) | 71.5 (8.1) | 71.7 (7.7) |
| **Sex, female, n (%)** | 30 (44.8) | 41 (47.7) | 152 (56.5) | 189 (64.5) | 1,118 (57.1) |
| **Region, n (%)**  Western Europe and Australia  North America  Other | 35 (52.2)  32 (47.8)  0 (0.0) | 39 (45.3)  44 (51.2)  3 (3.5) | 81 (30.1)  30 (11.2)  158 (58.7) | 88 (30.0)  35 (11.9)  170 (58.0) | 778 (39.7)  501 (25.6)  680 (34.7) |
| **Race, n (%)**  Asian  White  Other/unknown | 0 (0.0)  64 (95.5)  3 (4.5) | 1 (1.2)  82 (95.3)  3 (3.5) | 13 (4.8)  219 (81.4)  37 (13.8) | 16 (5.5)  241 (82.3)  36 (12.3) | 236 (12.0)  1,621 (82.7)  102 (5.2) |
| **Ethnic group, n (%)**  Hispanic or Latino  Not Hispanic or Latino  Not stated/unknown | 7 (10.4)  58 (86.6)  2 (3.0) | 4 (4.7)  81 (94.2)  1 (1.2) | 102 (37.9)  167 (62.1)  0 (0.0) | 103 (35.2)  189 (64.5)  1 (0.3) | 341 (17.4)  1,605 (81.9)  13 (0.7) |
| **Years of education, mean (SD)** | 15.0 (3.9) | 14.4 (3.6) | 13.3 (4.0) | 12.7 (3.9) | 13.4 (4.0) |
| ***APOE ε4* allele, n (%)**  *0 ɛ4*  *1 ɛ4*  *2 ɛ4* | 23 (34.3)  34 (50.7)  10 (14.9) | 25 (29.1)  39 (45.3)  22 (25.6) | 98 (36.4)  126 (46.8)  45 (16.7) | 120 (41.0)  127 (43.3)  46 (15.7) | 651 (33.2)  972 (49.6)  336 (17.2) |
| **Diagnosis at baseline, n (%)**  MCI due to AD  Mild AD dementia | 41 (61.2)  26 (38.8) | 54 (62.8)  32 (37.2) | 150 (55.8)  119 (44.2) | 151 (51.5)  142 (48.5) | 1,073 (54.8)  886 (45.2) |
| **CDR-SB, mean (SD)** | 3.9 (1.7) | 3.6 (1.6) | 3.59 (1.53) | 3.76 (1.61) | 3.65 (1.60) |
| **MMSE, mean (SD)** | 23.4 (3.3) | 24.2 (3.4) | 24.0 (3.0) | 23.7 (3.2) | 23.6 (3.1) |

APOE apolipoprotein E, PET positron emission tomography, SD standard deviation
